# The Effect of Dexmedetomidine on Oxidative Stress during Pneumoperitoneum

**DOI:** 10.1155/2014/760323

**Published:** 2014-01-08

**Authors:** Bahanur Cekic, Sukran Geze, Gulsum Ozkan, Ahmet Besir, Mehmet Sonmez, S. Caner Karahan, Ahmet Mentese

**Affiliations:** ^1^Department of Anesthesiology and Critical Care, Faculty of Medicine, Karadeniz Technical University, 61080 Trabzon, Turkey; ^2^Department of Nephrology, Faculty of Medicine, Karadeniz Technical University, 61080 Trabzon, Turkey; ^3^Department of Hematology, Faculty of Medicine, Karadeniz Technical University, 61080 Trabzon, Turkey; ^4^Department of Biochemistry, Faculty of Medicine, Karadeniz Technical University, 61080 Trabzon, Turkey

## Abstract

*Purpose*. This study was intended to investigate the effect of dexmedetomidine on oxidative stress response in pneumoperitoneum established in rats. *Methods*. Animals were randomized into three groups, group S: with no pneumoperitoneum, group P: with pneumoperitoneum established, and group D: given 100 mcg intraperitoneal dexmedetomidine 30 min before establishment of pneumoperitoneum. Plasma total oxidant status (TOS), total antioxidant status (TAS), and oxidative stress index (OSI) activity were measured 30 min after conclusion of pneumoperitoneum. *Results*. The mean TOS level was significantly higher in group P than in the other two groups, and the TOS level was significantly higher in group D than in group S (*P* < 0.05). Plasma TAS level was found to be lower in group P than in the other two groups, and the TAS level was lower in group D than in group S (*P* < 0.05). Consequently, the OSI was significantly higher in group P than in groups D and S (*P* < 0.05). *Conclusions*. Ischemia-reperfusion phenomenon that occurs during pneumoperitoneum causes oxidative stress and consumption of plasma antioxidants. Dexmedetomidine decreases oxidative stress caused by pneumoperitoneum and strengthens the antioxidant defense system.

## 1. Introduction

Laparoscopic surgery is performed widely because it causes less tissue trauma associated with shorter healing time compared with open surgery. Nevertheless, concerns regarding systemic complications and pathophysiology are still being investigated [1].

Clinical and experimental studies have established that the increase in intra-abdominal pressure that develops depending on the degree of pneumoperitoneum during laparoscopic surgery can cause hypoperfusion of intra-abdominal organs [2–6]. Increases in ischemia and the oxidative stress response were observed with pneumoperitoneum-dependent impairment of splenic perfusion [7–11]. After desufflation, reperfusion injury occurred with the fall in intra-abdominal pressure [3, 12].

Reactive oxygen products (ROS) like superoxide and hydroxyl radicals released during reperfusion are considered to contribute to reperfusion injury, because excessive ROS and their toxic products cause DNA damage, lipid peroxidation, and mitochondrial membrane and cell damage [13, 14]. Therefore ROS is considered a significant mediator of tissue injury. ROS is strictly controlled by complex antioxidants containing enzymes like superoxide dismutase, catalase, and glutathione peroxide [15, 16]. Free radicals formed as a result of pneumoperitoneum cause plasma antioxidants to decrease [17]. Thus one of the main results of ischemia-reperfusion due to pneumoperitoneum is the disturbance of the balance between the oxidative and antioxidative systems. The imbalance is defined as oxidative stress [18]. The severity of oxidative stress is determined by the measurement of total oxidant status (TOS) and consumed antioxidant status [17].

Various agents have been used in animal studies to protect organs from ischemia-reperfusion induced oxidative stress. Although these agents have been recommended for the protection of organs during various pathological conditions and surgical procedures in humans, their development and approval for clinical use are a lengthy process. One agent approved for clinical use that has been shown to protect the kidney against ischemia-reperfusion injury [19] is dexmedetomidine, a selective and potent *α*2-adrenoceptor agonist. It is frequently used for anesthesia in daily practice and for sedation, anxiolysis, and analgesia in the intensive care unit.

In this experimental study we aimed to investigate the effect of dexmedetomidine on oxidative stress in the ischemia-reperfusion injury due to pneumoperitoneum. We planned to use plasma total oxidant status (TOS), total antioxidant status (TAS), and oxidative stress index (OSI) parameters for determining the effect.

## 2. Materials and Methods

This study was approved by the ethics committee of our university and was performed in compliance with the National Institutes of Health Guidelines for the Care and Use of Laboratory Animals.

### 2.1. Animal Preparation

Twenty-four adult female Sprague-Dawley rats weighing 250–300 g were used in this study. The animals were kept in a windowless, light-controlled environment at 20 ± 2°C and were allowed free access to food and water. They were fasted for one night before the experiment. The animals were anesthetized with 50 mg/kg ketamine (Ketalar; Parke Davis, Berlin, Germany) and 20 mg/kg xylazine (Rompun; Bayer, Leverkusen, Germany), and were placed in a supine position on an operating table. The tail vein was cannulated with a 24 G intravenous catheter. After the tracheal region was cleaned, the trachea was isolated with a midline incision and cannulated with a 16 G intravenous catheter. Mechanical ventilation was initiated in volume-controlled mode with a respiratory frequency of 40/min, tidal volume of 10 mL/kg, inspirium/expirium ratio of 1 : 1, and fractional inspiratory oxygen concentration (FiO_2_) of 1.0. Spontaneous respiration was suppressed with intravenous pancuronium (1 mg/kg).

### 2.2. Experimental Protocol

Following an initial stabilization period, the animals were randomized into three groups (*n* = 8 in each): group S (sham group), no pneumoperitoneum was established; group P (pneumoperitoneum group), 60 min pneumoperitoneum was established under 12 mm Hg pressure; and group D (ischemia-reperfusion/dexmedetomidine treatment group), intraperitoneal dexmedetomidine (100 *μ*g) was administered 30 min before abdominal insufflation to establish 60 min pneumoperitoneum under 12 mm Hg pressure.

### 2.3. Establishment of Pneumoperitoneum

Pneumoperitoneum was established by inserting an 18 G intravenous catheter into the abdominal right lower quadrant of the peritoneal cavity and insufflating the abdomen with CO_2_ to a pneumoperitoneal pressure of 12 mm Hg. The intra-abdominal pressure was maintained for 60 min with an electronic laparoflator (Karl-Starz GmbH, Tutlingen, Germany).

### 2.4. Blood Samples

Blood samples were obtained in tubes containing 3.8% sodium citrate as an anticoagulant. Plasma and serum were separated by centrifugation at 3000 rpm for 10 min. The serum and plasma samples were kept at −80°C until biochemical analysis.

### 2.5. Measurements

Blood plasma total antioxidant status (TAS) and total oxidant status (TOS) were measured 30 min after desufflation.

Plasma TOS was determined using a method as previously described by Erel [20] and is expressed as *μ*mol H_2_O_2_ equivalent/L. Serum TAS was determined using an automated measurement method developed by Erel [21] and is expressed as mmol Trolox equivalent/L. The OSI was calculated from the TOS and TAS values: OSI = [(TOS, *μ*mol H_2_O_2_ equivalent/L)/(TAS, *μ*mol Trolox equivalent/L)] × 100 [22].

### 2.6. Statistical Analysis

Statistical analysis was performed using two-way and three-way ANOVA. All values are expressed as means ± SD. Significance was set at *P* < 0.05.

## 3. Results

All rats survived until the end of the experiment. Body weight was similar among the groups (202.62 ± 28.86, 211.00 ± 14.45, and 212.87 ± 15.71 g in groups S, D, and P, resp.).

**Figure 1:**
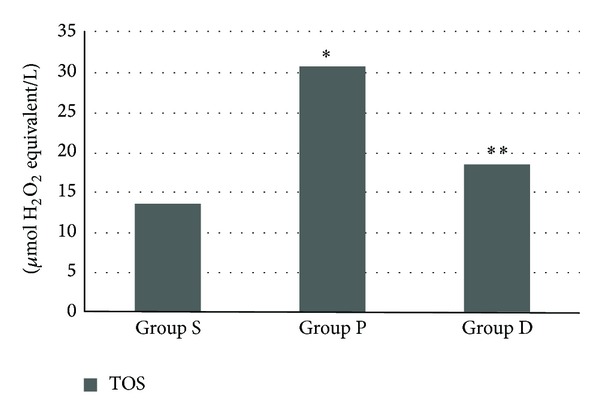
Total oxidant status (TOS) levels (group S: 13.56 ± 1.72, group P: 30.67 ± 3.06, and group D: 18.43 ± 1.93). *P < 0.0001 group P when compared with D and S groups and **P < 0.01 group D when compared with group S.

**Figure 2:**
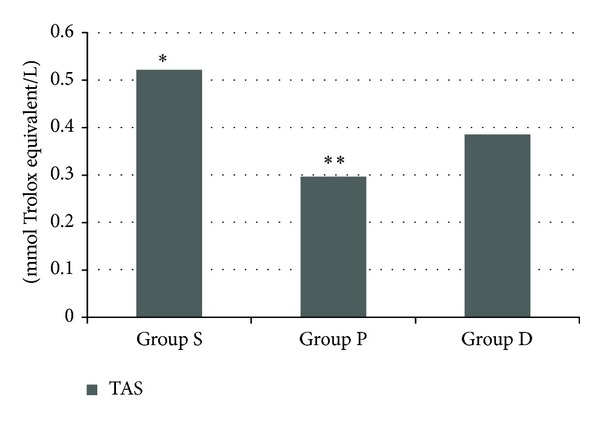
Total antioxidant status (TAS) levels (group S: 0.52 ± 0.49, group P: 0.29 ± 0.06, and group D: 0.38 ± 0.05). *P < 0.001 group S when compared with D and C groups and **P < 0.015 group P when compared with group D.

**Figure 3:**
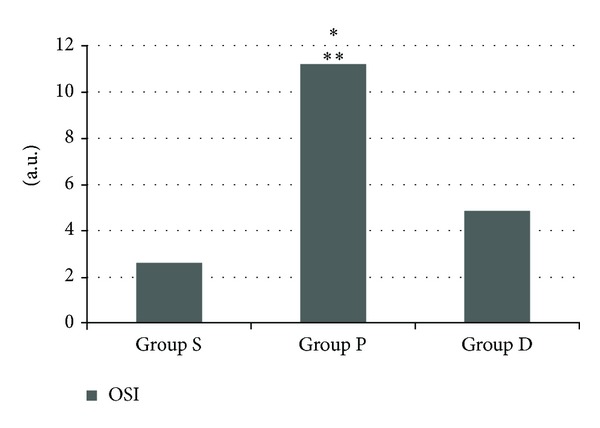
Oxidative stress index (OSI) levels (group S: 2.61 ± 0.48, group P: 11.2 ± 4.90, and group D: 4.86 ± 0.89). *P < 0.0001 group P when compared with groups S and **P < 0.001 group P when compared with group D.

The mean TOS level was significantly higher in group P than in the other two groups (*P* < 0.0001), and the TOS level was significantly higher in group D than in group S (*P* < 0.01) (Figure 1). Plasma TAS level was found to be lower in pneumoperitoneum group than in groups S and D (*P* < 0.0001 and < 0.015, resp.), and TAS level was lower in D group than in group S (*P* < 0.0001) (Figure 2). Consequently, the OSI was significantly higher in group P than in groups D and S (*P* < 0.001 and < 0.0001, resp.) (Figure 3).

## 4. Discussion

In our study we determined that plasma TOS level and OSI score decreased, and antioxidant defense system strengthened with dexmedetomidine administration before pneumoperitoneum in rats. We observed that dexmedetomidine prevented oxidative damage caused by pneumoperitoneum.

The free oxygen radicals are known to be released during this reperfusion period and have been proposed as important mediators of tissue injury [23]. Free oxygen radicals induced lipid peroxidation associated with a decrease in plasma antioxidant capacity [7]. Reactive oxygen radicals play a vital role in the injury caused by ischemia-reperfusion. Many studies for animals [2, 7, 11] and human beings [24, 25] have investigated ischemia-reperfusion injury caused by pneumoperitoneum. In these studies oxidative stress and lipid peroxidation markers were evaluated with measurements of different markers like thiobarbituric acid reactive substances (TBARS), endogen antioxidant level, and histological findings to obtain protein carbonyl and protein sulfhydryl content [26]. These measurements showed that pneumoperitoneum may cause an increase in the oxidative stress response and ROS may cause damage to lipids and proteins during oxidative stress.

In this study we observed a significant increase in total plasma level after pneumoperitoneum. Hydrogen peroxide and other peroxide derivatives are produced biologically in organisms, but in some pathological cases it is produced at high levels [20]. In particular high oxygen concentration in the circulation during the reperfusion causes the formation of free radicals. Baysal et al. determined that free radicals formed during laparoscopy caused oxidative stress in children, increased plasma oxidant status and OSI level after pneumoperitoneum, and decreased total antioxidant status [17]. Similarly we observed an increase in plasma oxidative stress index and a decrease in total antioxidant status level after pneumoperitoneum deflation.

To reverse the oxidative stress caused by pneumoperitoneum, various pharmacological agents and protective methods have been tested [12, 23, 27, 28]. Ates et al. discovered that erythropoietin administration before pneumoperitoneum caused a significant decrease in LDH, TNF-*α*, and MDA levels [27]. Imamoğlu et al. showed that melatonin administration before pneumoperitoneum insufflation and deinsufflation decreased kidney MDA level [23]. Similarly zinc, pentoxifylline, and NAC administration to renal ischemia-reperfusion injury caused by laparoscopy decreases kidney tissue MDA level [28].

Dexmedetomidine, an *α*2-adrenergic agonist, has been shown experimentally to prevent ischemia-reperfusion injury by producing vasodilation and is frequently used in anesthesia and intensive care practice [19, 29]. Apart from sedative, analgesic characteristics of dexmedetomidine it has been shown that it has the ability to relieve the lung injury caused by renal ischemia-reperfusion [30]. Dexmedetomidine provides local protection in ischemic kidney and following that generates anti-inflammatory effect by decreasing systemic accumulation of cytokines. This effect is determined with evidence at preclinical and clinical level [19, 30].

Positive effect of dexmedetomidine has been shown on ischemia-reperfusion experimental models where oxidative injury plays a significant role. But systemic effect on oxidative stress is not shown yet.

In the light of findings of our study we determined that oxidative stress response decreases and antioxidant defense system strengthens with the administration of dexmedetomidine before pneumoperitoneum. We determined that plasma total oxidant status and OSI level were lower and total antioxidant status level was higher in groups to which dexmedetomidine was administered before pneumoperitoneum. We observed that dexmedetomidine can play a role in preventing the oxidative injury caused by pneumoperitoneum.

Laparoscopic procedure can be used widely for patients from different age groups in early future. Administration of laparoscopic procedure can increase the severity of ischemia-reperfusion injury in old patients with malignity and cardiovascular problems. Therefore dexmedetomidine as anesthetic agent may be preferred as protective agent in preventing adverse effects of oxidative injury.
